# Constraining Genome-Scale Models to Represent the Bow Tie Structure of Metabolism for ^13^C Metabolic Flux Analysis

**DOI:** 10.3390/metabo8010003

**Published:** 2018-01-04

**Authors:** Tyler W. H. Backman, David Ando, Jahnavi Singh, Jay D. Keasling, Héctor García Martín

**Affiliations:** 1 Joint BioEnergy Institute, 5885 Hollis Street, Emeryville, CA 94608, USA; tbackman@lbl.gov (T.W.H.B.); david.ando@lbl.gov (D.A.); jahnavis@lbl.gov (J.S.); jdkeasling@lbl.gov (J.D.K.); 2 Agile BioFoundry, 5885 Hollis Street, Emeryville, CA 94608, USA; 3 Biological Systems and Engineering Division, Lawrence Berkeley National Laboratory, Berkeley, CA 94720, USA; 4 QB3 Institute, University of California, Berkeley, CA 94720, USA; 5 Department of Bioengineering, University of California, Berkeley, CA 94720, USA; 6 Department of Computer Science, University of California, Berkeley, CA 94720, USA; 7 Department of Chemical and Biomolecular Engineering, University of California, Berkeley, CA 94720, USA; 8 Novo Nordisk Foundation Center for Biosustainability, Technical University of Denmark, 2970 Horsholm, Denmark

**Keywords:** genome scale models, ^13^C metabolic flux analysis, two-scale ^13^C metabolic flux analysis, flux balance analysis, stoichiometry, linear programming, cellular metabolism, simulated annealing

## Abstract

Determination of internal metabolic fluxes is crucial for fundamental and applied biology because they map how carbon and electrons flow through metabolism to enable cell function. ${}^{13}$C Metabolic Flux Analysis (${}^{13}$C MFA) and Two-Scale ${}^{13}$C Metabolic Flux Analysis (2S-${}^{13}$C MFA) are two techniques used to determine such fluxes. Both operate on the simplifying approximation that metabolic flux from peripheral metabolism into central “core” carbon metabolism is minimal, and can be omitted when modeling isotopic labeling in core metabolism. The validity of this “two-scale” or “bow tie” approximation is supported both by the ability to accurately model experimental isotopic labeling data, and by experimentally verified metabolic engineering predictions using these methods. However, the boundaries of core metabolism that satisfy this approximation can vary across species, and across cell culture conditions. Here, we present a set of algorithms that (1) systematically calculate flux bounds for any specified “core” of a genome-scale model so as to satisfy the bow tie approximation and (2) automatically identify an updated set of core reactions that can satisfy this approximation more efficiently. First, we leverage linear programming to simultaneously identify the lowest fluxes from peripheral metabolism into core metabolism compatible with the observed growth rate and extracellular metabolite exchange fluxes. Second, we use Simulated Annealing to identify an updated set of core reactions that allow for a minimum of fluxes into core metabolism to satisfy these experimental constraints. Together, these methods accelerate and automate the identification of a biologically reasonable set of core reactions for use with ${}^{13}$C MFA or 2S-${}^{13}$C MFA, as well as provide for a substantially lower set of flux bounds for fluxes into the core as compared with previous methods. We provide an open source Python implementation of these algorithms at https://github.com/JBEI/limitfluxtocore.

## 1. Introduction

Current limitations in quantitatively predicting biological behavior hinder our efforts to engineer biological systems to produce biofuels and other desired chemicals [1]. Determination of internal metabolic fluxes (i.e., the amount of metabolites traversing each biochemical reaction per unit time [2,3]) is a useful tool in this effort because they map how carbon and electrons flow through metabolism to enable cell function [3,4] and can produce actionable insights to increase biofuel production [5]. Several methods are available for calculating internal metabolic fluxes. Arguably, the most popular are Flux Balance Analysis (FBA) and ${}^{13}$C Metabolic Flux Analysis (${}^{13}$C MFA). FBA determines fluxes by using comprehensive genome-scale models and by assuming that cells closely follow an evolutionary principle of maximizing biomass. ${}^{13}$C MFA calculates fluxes by constraining small models of central carbon metabolism with the strong flux constraints obtained from ${}^{13}$C labeling experiments [6,7,8]. Proponents of each of these two techniques rarely combine them, except for a few exceptions (e.g., [9,10,11,12,13,14,15]). FBA and COnstraint Based Reconstruction and Analysis (COBRA) can predict all fluxes in a large genome scale model using an optimization principle, but do not directly constrain internal fluxes with high resolution experimental data. Conversely, ${}^{13}$C MFA models are well constrained by experimental data, but only measure a small number of central carbon metabolism fluxes, and do not model the full complexity and plasticity of a large metabolic network. However, metabolic engineering can benefit from uniting the advantages of both approaches: a method that provides fluxes for comprehensive genome-scale models as constrained by the very informative ${}^{13}$C labeling experimental data.

**Figure 1:**
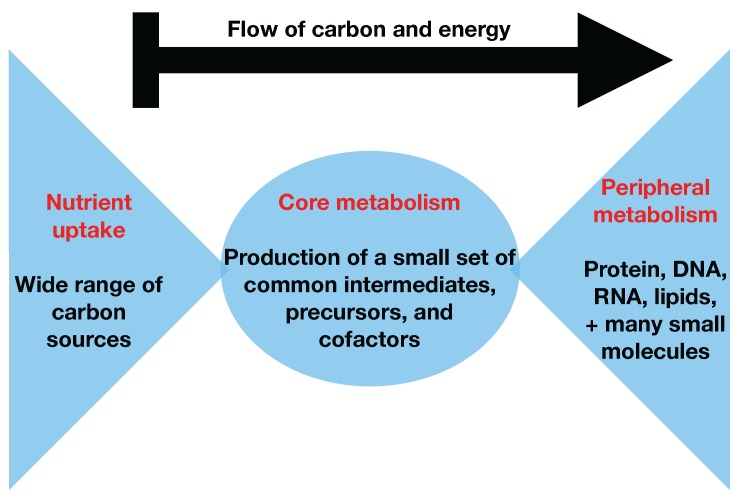
The bow tie structure of metabolism. The bow tie structure of metabolism describes how numerous different types of carbon sources (left side of figure) are funneled into a small set of precursor metabolites (middle of figure) in central carbon metabolism that are subsequently processed into a large set of peripheral metabolites (right side of figure) for the formation of biomass in the form of protein, DNA, RNA, cell wall, and other cellular components.

**Figure 2:**
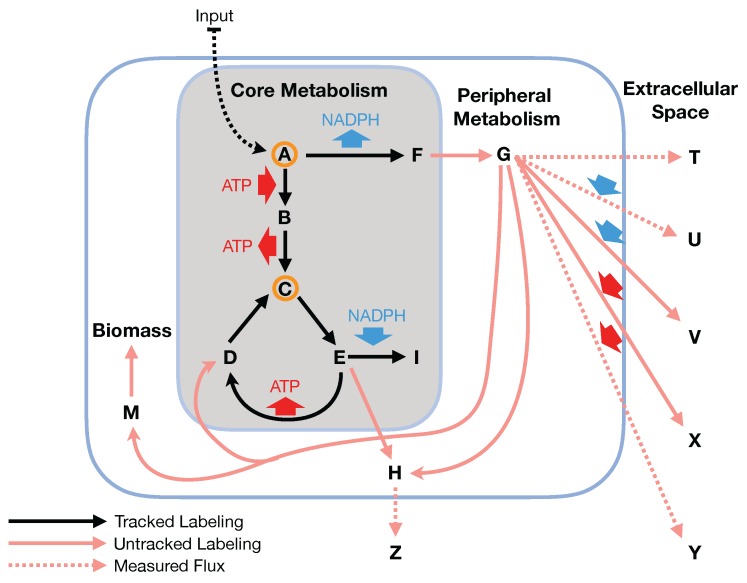
The bow tie approximation (a.k.a. two-scale approximation) assumes that flux flows from core to peripheral metabolism and there is a limited flow back. 2S-${}^{13}$C MFA is based on this assumption, providing fluxes constrained by ${}^{13}$C data for genome-scale models [23]. Under 2S-${}^{13}$C MFA, stoichiometric balances are taken into account for the full genome-scale model, but labeling originating from the ${}^{13}$C feed in the labeling experiments is only tracked for the core set of reactions responsible for the main fraction of metabolite labeling (grey box). The bow tie approximation implies that non-core (peripheral) metabolites do not directly affect core metabolite labeling. For this example, measured data involves the mass isotopomer distribution for metabolites A and C and extracellular fluxes for reactions producing metabolites T, U, Y and Z as well as labeled carbon (Input) uptake rate and Biomass growth rate. The core set involves reactions and metabolites in the grey box. The fit involves finding fluxes that best match the measured labeling and the values of the measured extracellular fluxes, where only the contribution of reactions inside the grey box is taken into account to fit the labeling of metabolites A and C. The metabolite balance, however, is global. In this way, the core fluxes are not overconstrained by (e.g.,) NADPH balance: any excess core NADPH can be balanced by the non-core fluxes that consume NADPH. Fluxes are determined for 2S-${}^{13}$C MFA in a single optimization problem instead of using the constraints from the ${}^{13}$C MFA problem to constrain an equivalent FBA problem as done by Kuepfer et al. [15]. This enables stoichiometric constraints from outside of the core set to influence the core fluxes in the solution. The 2S-${}^{13}$C MFA method provides a reliable base upon which to improve the design of engineered biological systems [5].

While ${}^{13}$C MFA has been performed at the genome scale for *E. coli* [16], it is a computationally expensive method and requires knowledge of all of the carbon transitions in the network. This knowledge is nontrivial to obtain systematically for any desired organism [17,18,19,20,21,22]. Two-scale ${}^{13}$C Metabolic Flux Analysis [23] (2S-${}^{13}$C MFA) is an alternative technique that constrains genome-scale models with ${}^{13}$C labeling experimental data. 2S-${}^{13}$C MFA constrains all fluxes in the genome-scale model simultaneously using stochiometric and ${}^{13}$C labeling constraints, but does so at two resolution scales: for core reactions, both stochiometric and ${}^{13}$C labeling constraints are used, whereas, for non-core reactions, only stochiometric constraints are used. 2S-${}^{13}$C MFA is meant to obtain the same results as genome-scale ${}^{13}$C MFA if we assume that flux flows from core to peripheral metabolism and there is limited flow back. This assumption is named the two-scale or bow tie approximation (Figure 1 and  Figure 2) and stems from the bow tie structure of cellular metabolism, a universally conserved product of evolution [24,25]. Hence, in this paper, we will refer to it indistinctly as the bow tie approximation or the two-scale approximation. The bow tie structure entails, as shown in Figure 1, that almost all carbon and energy sources are converted through central carbon metabolism pathways into a set of twelve precursor metabolites (glucose-6-phosphate, fructose-6-phosphate, ribose-5-phosphate, erythrose-4-phosphate, glyceraldehyde-3-phosphate, 3-phosphoglycerate, phosphoenol-pyruvate, pyruvate, acetyl-CoA, 2-oxoglutarate, succinyl-CoA, and oxaloacetate), which are the building blocks of most cellular components and natural products synthesized by cells [26]. Hence, if carbon sources are included in the core metabolism, the bow tie structure implies that flux flows from core to peripheral metabolism and there is limited flow back. This bow tie approximation is experimentally justified by the fact that traditional ${}^{13}$C MFA, using only core metabolism models, can convincingly explain labeling patterns for amino acids and intracellular metabolites for model organisms [27,28,29,30], and by experimentally verified metabolic engineering predictions using 2S-${}^{13}$C MFA [5].

In this paper, we formalize the bow tie approximation (a.k.a. two-scale approximation) in terms of genome-scale models by providing improved and systematic methods to (1) constrain fluxes in accordance with this approximation and (2) determine the boundaries of core metabolism. In the context of genome-scale models, the bow tie approximation is implemented by setting the upper bound of all reactions with products in core metabolism to zero, or the lowest value consistent with the observed growth rate (see “Limit Flux to Core” step in Figure 2 of [23] and Figure 2). The previously published 2S-${}^{13}$C MFA [23] implementation of the “Limit Flux to Core” step used an algorithm that limited the upper bounds of fluxes into the core using an inefficient and ad hoc process which relied on trial and error, arbitrary cutoff values, and sequential execution. Here, we present an improved method which uses linear optimization to find the minimum flux bounds into the core, which is computationally more efficient, and also more biologically relevant, as we identify the lowest flux into core metabolism consistent with observed experimental data. Minimizing the flux of reactions into the core through linear programming is complicated by the reversible nature of some reactions which cross the core boundary. We solve this problem by constructing a minimization procedure which considers only the unidirectional component of each boundary flux that has products in core metabolism.

For a given core, a metric to quantitatively determine to what degree the bow tie approximation holds, is the sum of fluxes into the core (zero for a perfect case of the bow tie structure). Therefore, we also introduce a Simulated Annealing algorithm to computationally explore the space of alternate core metabolism reaction sets, minimizing the sum of fluxes flowing into core metabolism. This process can automatically identify an improved core (displaying less total flux into core) which has more or less reactions as needed to better satisfy the bow tie approximation and be more suitable for subsequent ${}^{13}$C MFA or 2S-${}^{13}$C MFA modeling.

## 2. Materials and Methods

### 2.1. Old Limit Flux to Core Algorithm

Under the bow tie approximation, non-core reactions in the periphery do not contribute directly to the labeling of core metabolites because carbon precursors are assumed to flow from core metabolism into peripheral metabolism and not to flow back (Figure 2). This approximation is implemented in terms of a genome-scale model by limiting to zero the flux of reactions flowing into core metabolism. Hence, the first step in 2S-${}^{13}$C MFA consists of taking each non-core reaction that has a product in core metabolism and setting the upper bound to zero. However, it may be the case that this extra constraint makes it impossible to meet the measured growth rate, as determined by solving the corresponding FBA problem. In that case, setting the upper bound to a fraction of the glucose uptake rate is tested (first 0.05 and then 0.2 by default). Since the labeling of core metabolism can be impacted by reversible reactions with reactants included in the core set as well, we cover this case by limiting the lower bound of the reaction to zero or the lowest value that permits growth (e.g., biomass flux). The impact of the reactions that could not be set to zero is checked later in the 2S-${}^{13}$C MFA procedure through External Labeling Variability Analysis [23]. The input for the “Limit Flux to Core” algorithm is the genome-scale model along with a set of reactions that define core metabolism, a cellular growth rate, and a set of exchange fluxes including the carbon source uptake rate. The output consists of the genome-scale model with lower and upper bounds modified by this “Limit Flux to Core” procedure.

A detailed description of the old Limit Flux to Core algorithm be found in the pseudo code Algorithms 1 and 2. This algorithm was previously published as part of the 2S-${}^{13}$C MFA method [23], and is implemented in the jQMM software tool [31]. The set of “boundary reactions” which can potentially alter ${}^{13}$C labeling of core metabolites is determined for both Algorithms 2 and 3 using Algorithm 1. This requires, for any given core, first specifying a set of “currency metabolites” which participate in core reactions, but (based on known atom transitions) cannot contribute carbon to any of the simulated metabolites in a 2S-${}^{13}$C MFA or ${}^{13}$C MFA model (e.g., ATP, NADH). Our software includes a pre-determined list of suggested “currency metabolites”; however, most popular software tools for 2S-${}^{13}$C MFA or ${}^{13}$C MFA, including the jQMM library  [31], can compute these directly from a set of core reaction atom transitions. Reactions which feed only currency metabolites into core metabolism are excluded from the set of boundary reactions for subsequent flux minimization.

**Algorithm 1** Identify core boundary reactions.1:Function CoreBoundary(genomeScaleModel, coreReactionSet, currencyMetaboliteSet):2: boundaryReactionSet = emptySet()3: For reaction in coreReactionSet:4:  For reactant in reaction.reactants and not in currencyMetaboliteSet: **# reactants includes products if reversible**5:   For otherReaction in genomeScaleModel.reactions and not in coreReactionSet:6:    If reactant in otherReaction.products: **# products includes reactants if reversible**7:     boundaryReactionSet.add(otherReaction)8: return boundaryReactionSet

**Algorithm 2** Previous Limit Flux to Core Algorithm.  1:Function LimitFluxToCore(genomeScaleModel, coreReactionSet, currencyMetaboliteSet, carbonUptakeFlux):  2: boundaryReactionSet = CoreBoundary(genomeScaleModel, coreReactionSet, currencyMetaboliteSet)  3: limits = [0, 0.05, 0.2] * carbonUptakeFlux  4: fluxLimitsTowardsCoreResults = emptySet()  5: for reaction in boundaryReactionSet:  6:  i = 0  7:  reaction.fluxLimit = limits[i] **# this is the flux limit in the direction producing core metabolites**  8:  While hasNoFBASolution(genomeScaleModel):  9:   i = i + 110:   reaction.fluxLimit = limits[i]11:  fluxLimitsTowardsCoreResults.add(reaction: reaction.fluxLimit)12: return fluxLimitsTowardsCoreResults

### 2.2. New Limit Flux to Core algorithm

Algorithm 2 is robust and has allowed for the effective measurement of fluxes through the 2S-${}^{13}$C modeling approach. Upon inspection, Algorithm 2 is fundamentally a sequential routine which tests boundary fluxes one by one to determine if they can be zeroed, and which sets flux bounds to arbitrary limits when this testing fails. We present a faster and improved algorithm for limiting the flux to the core from the periphery, which involves a minimization procedure for flux into the core that is consistent with the observed growth rate of the organism. This is achieved by Algorithm 3 using a methodology where all reactions are split into a pair of unidirectional fluxes (e.g., a forward and reverse flux as separate components), and the sum of all unidirectional fluxes into the core is minimized in a single step through linear programming. This has several key advantages as demonstrated in the results section below: (1) the limits of flux into the core are substantially lower than those obtained with Algorithm 2; (2) the run time for computing these limits is reduced by several orders of magnitude, as only a single linear optimization is performed instead of hundreds; and (3) there is no arbitrary bias introduced by the order in which individual reactions are minimized, as was the case in Algorithm 2.

The “minimize flux into core” problem is solved by Algorithm 3 using linear programming with the following constraints:

(1) $$\mathrm{Minimize}\sum_{j\in J}c_{j}v_{j},$$

Subject to:

(2) $$\sum_{i,j}S_{ij}v_{j}=0\;\;\forall i\in I,j\in J,$$

(3) $$lb_{j}\leq v_{j}\leq ub_{j}\;\;\forall j\in J,$$

where:
*I*: Set of all metabolites;*J*: Set of all fluxes where reversible reactions are treated as two unidirectional reactions;$c_{j}$: Vector selecting unidirectional flux components with products in core (e.g., from reactions with non-currency metabolite products in the core, as well as reversible reactions with reactants in core);$S_{ij}$: Stoichiometry matrix;$v_{j}$: Flux value of reaction j∈J;$ub_{j},lb_{j}$: Upper and lower bounds for *j*.

**Algorithm 3** New Limit Flux to Core algorithm.1:Function LimitFluxToCore(genomeScaleModel, coreReactionSet, currencyMetaboliteSet):2: boundaryReactionSet = CoreBoundary(genomeScaleModel, coreReactionSet, currencyMetaboliteSet)3: genomeScaleModel.FBAobjective = $\sum_{r\in boundaryReactionSet}$ r.FluxLimitTowardsCore **# FluxLimitTowardsCore is the unidirectional flux components with a product in core metabolism, this step is equivalent to the sum in Equation (1)**4: fluxes = FBAminimization(genomeScaleModel)5: fluxLimitsTowardsCoreResults = emptySet()6: for flux in fluxes:7:  if flux.reaction in boundaryReactionSet:8:   fluxLimitsTowardsCoreResults = fluxLimitsTowardsCoreResults.add(flux.reaction: flux.FluxTowardsCore)9: return fluxLimitsTowardsCoreResults

### 2.3. Calculating Fluxes Using the Different “Limit Flux to Core” Algorithms

We used the jQMM library [31] to calculate genome scale fluxes for *E. coli* using both the old “Limit flux to core” procedure given by Algorithm 2 and via the new “Limit flux to core” procedure as given by Algorithm 3. For calculating fluxes using the old “Limit flux to core” algorithm, the jupyter notebook “A4: FluxModels demo” was used. This notebook is included in the public release of the jQMM library found at http://github.com/JBEI/jqmm. Genome scale fluxes using the new “Limit flux to core” algorithm were also calculated using the “A4: FluxModels demo”, except that the limitFlux2Core flag was set to False when calculating fluxes via the findFluxesRanges command and flux bounds into the core were calculated using the example Jupyter notebook included in the public release of the new limit flux to core algorithm at http://github.com/JBEI/limitfluxtocore.

### 2.4. Simulated Annealing Core Boundary Improvement Algorithm

To successfully perform 2S-${}^{13}$C MFA, or ${}^{13}$C MFA on a subset of the metabolic reactions in a given species, it is necessary to first identify the set of core reactions where the bow tie approximation is valid under the tested experimental conditions. For common model organisms, previously published literature will often include a reasonable set of core reactions suitable for common laboratory cell culture conditions. In many cases, these previously published core boundaries were painstakingly manually curated by iteratively expanding the core boundaries until a set of reactions is found which constitute a model that can explain experimental data within the bounds of experimental error [23].

However, this labor intensive process must be repeated when modeling a new species, or a previously studied species under radically altered growth conditions. Here, we present a new method, Algorithm 4, which automates this process by systematically exploring the core boundary space with Simulated Annealing, and computing the sum of fluxes into core metabolism using Algorithm 3 at each iteration [32]. For a given set of core reactions and experimentally measured exchange fluxes, the sum of minimized fluxes into the core represents the “energy” (which we are trying to minimize) in Simulated Annealing terms. The “energy landscape” is systematically explored by iteratively and randomly adding or removing reactions from the boundaries of the core metabolism set, and checking the new “energy” at each step. An improved (e.g., lower energy) core set is always accepted, whereas an inferior (e.g., higher energy) core set is conditionally accepted at each iteration with a probability computed by Equation (4). Occasionally accepting an inferior solution allows the Simulated Annealing algorithm to escape local minima, and find a better solution which requires first traversing an inferior solution.

(4) $$P\left(acceptance|t,E_{old},E_{new}\right)=\left\{\begin{array}{cc} e^{\frac{E_{old}-E_{new}}{t}}, & E_{old}<E_{new},\\ 1, & E_{old}\geq E_{new}, \end{array}\right.$$

where:
$E_{old}$: Energy (in units of metabolic flux) of the previous iteration;$E_{new}$: Energy (in units of metabolic flux) of the current iteration;*t*: Temperature of the current iteration (in units of metabolic flux) per the user specified annealing schedule.

The temperature is gradually and iteratively lowered over the course of the simulation, from a high initial starting temperature to a low final temperature following an exponentially decaying annealing schedule. This annealing schedule provides an initial high temperature where the algorithm can efficiently escape local minima, while a long and slow “cooling” process encourages eventual settling in a new local minima. As Simulated Annealing is stochastic, a different sequence of random numbers can alter the final solution obtained. Therefore, we recommend running the algorithm multiple times with an array of different random number generator seeds, in order to find the best solution from a set of many simulations.

The computational overhead of finding improved core boundaries with Algorithm 4 can be substantially lowered by starting with a reasonable initial set of core reactions as a starting point. Two possible methods for computing a reasonable starting set of core reactions include: (1) including all reactions which are shared in common between the current species or strain and the phylogenetically closest previously published species with a known valid ${}^{13}$C MFA core reaction set; (2) by systematically testing a genome scale model with Flux Balance Analysis under randomized growth conditions for a common set of key core reactions (e.g., using the method published by Almas et al. [33]). Algorithm 4 also provides a minimum overlap parameter, which will limit the number of reactions that can be removed from the starting core set, as well as a maximum size parameter that limits the number of new reactions added. This is an important feature because a trivially “perfect” core with no flux from peripheral metabolism could include, for example, just the carbon uptake exchange flux and no other reactions, or, conversely, it could include the entire genome scale model. The minimum overlap parameter can ensure that most or all experimentally measured core metabolites are retained in the model, while the maximum size parameter avoids expanding the core into a size that is computationally prohibitive to model with ${}^{13}C$ MFA.

**Algorithm 4** Simulated annealing core improvement algorithm.  1:Function AnnealCore(genomeScaleModel, InitialCoreReactionSet, AnnealingSchedule, minimumOverlap, maximumSize, currencyMetaboliteSet):  2: currentSet = InitialCoreReactionSet  3: oldBoundaryFluxes = LimitFluxToCore(genomeScaleModel, currentSet, currencyMetaboliteSet)  4: oldEnergy = ∑ oldBoundaryFluxes  5: for temperature in AnnealingSchedule:  6:  newSet = MoveCore(genomeScaleModel, currentSet, InitialCoreReactionSet, minimumOverlap, maximumSize)  7:  newBoundaryFluxes = LimitFluxToCore(genomeScaleModel, newset, currencyMetaboliteSet)  8:  newEnergy = ∑ newBoundaryFluxes  9:  if newEnergy < oldEnergy:10:   currentSet = newSet11:  else if $Exp\left(\frac{oldEnergy-newEnergy}{temperature}\right)>Rand\left(0,1\right)$: **# here Rand returns a random floating point number between 0 and 1**12:   currentSet = newSet13:  oldEnergy = newEnergy14: return currentSet15: 16:Function MoveCore(genomeScaleModel, currentSet, InitialCoreReactionSet, minimumOverlap, maximumSize):17: if (Rand(0,1) > 0.5) or (length(currentSet) ≥ maximumSize): **# here Rand returns a random floating point number between 0 and 1**18:  randomReaction = selectRandomElement(currentSet)19:  newCore = currentSet.remove(randomReaction)20:  if IsConnectedComponent(newCore, genomeScaleModel): **# check if all reactions are connected to each other**21:   overlap = length(InitialCoreReactionSet.intersection(newCore))/length (InitialCoreReactionSet)22:   if overlap ≥ minimumOverlap:23:    return newCore24:   else:25:    return currentSet26:  else:27:   return currentSet28: else:29:  boundaryReactionSet = CoreBoundary(genomeScaleModel, currentSet, currencyMetaboliteSet)30:  return currentSet.add(selectRandomElement(boundaryReactionSet))

### 2.5. Implementation of Simulated Annealing and Limit Flux to Core Algorithms

We provide an open source Python implementation of Algorithms 1, 3 and 4 at https://github.com/JBEI/limitfluxtocore. This implementation leverages the capabilities of the COBRApy Python library for importing and analyzing genome scale models, as well as for performing linear optimization using an OptLang objective function [34,35]. Our implementation of Algorithm 4 makes use of the general purpose simanneal Python library for Simulated Annealing (https://github.com/perrygeo/simanneal).

## 3. Results

### 3.1. Minimizing Flux into Core Metabolism

**Figure 3:**
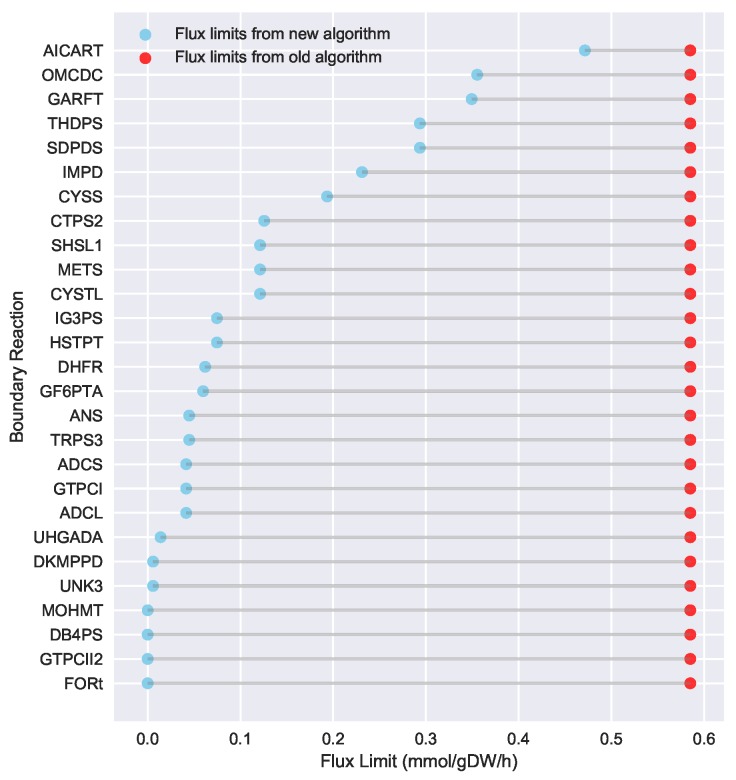
Flux bounds for core boundary reactions computed via the new Limit Flux to Core algorithm (blue) are much lower than those calculated with the old one (red). Our goal is not only to set to zero all reactions that can be zeroed, but also find the maximum allowable upper bound for those that cannot be zeroed. Upper flux bounds are shown for all reactions outside of core metabolism with products in core metabolism for the iJR904 *E. coli* genome scale model, using exchange and biomass fluxes from the previously published Toya et al. wild-type 5 h data [36,37]. The set of core reactions was taken from the Toya et al. (2010) example included with the jQMM software tool [31]. Flux bounds for the old Algorithm 2 are shown in red, whereas improved much lower flux bounds for the new Algorithm 3 are shown in sky-blue. Boundary reactions with fluxes into core metabolism that could be set to zero are not shown. Using the new algorithm, much lower bounds for reactions flowing into the core can be obtained. This limits the amount of flux flowing into the core and, hence, translates into better fulfillment of the bow tie approximation.

To evaluate the ability of Algorithm 3 to identify lower flux bounds into core metabolism than Algorithm 2, we ran both algorithms on the iJR904 *E. coli* genome scale model, using exchange and biomass fluxes from the previously published Toya et al. wild-type 5 h sample data [36,37]. We used a standard set of 127 core *E. coli* reactions that has been previously published as part of the jQMM software tool [31]. These core reactions result in a set of 219 “boundary reactions” that either produce a product consumed by core metabolism, or are reversible and include a reactant consumed by core metabolism. For generating the boundary reaction list, we excluded “currency metabolites” such as NADPH, which participate in core reactions without exchanging carbon. Both Limit Flux to Core algorithms identified a set of 27 reactions with products in core metabolism where the unidirectional component producing a product in core metabolism could not be set to zero. For the old algorithm (Algorithm 2), the minimized upper flux bound found for each was 0.585 mmol·gDW${}^{-1}$·h${}^{-1}$, resulting in a maximum sum of fluxes into core metabolism of approximately 15.80 mmol·gDW${}^{-1}$·h${}^{-1}$. The new algorithm presented here (Algorithm 3) was able to identify substantially lower flux limits for these same reactions, resulting in a much smaller maximum sum of fluxes into core metabolism of approximately 3.19 mmol·gDW${}^{-1}$·h${}^{-1}$. Figure 3 shows a comparison between the flux bounds identified with each method.

**Figure 4:**
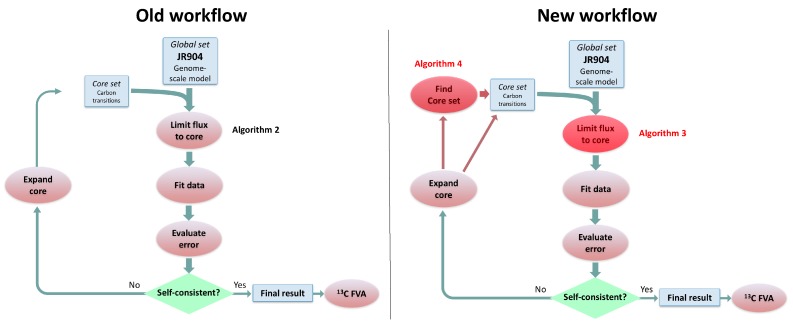
Comparison of old algorithm workflow for 2S-${}^{13}$C MFA versus the new workflow using the algorithms presented here. The left panel represents the old workflow (Figure 2 in Garcia Martin et al. [23] and the right panel portrays the new workflow, where the new algorithms are shown with a red overlay. Algorithm 2 presented in this paper represents the old “Limit flux to core” algorithm that is replaced by Algorithm 3 in this paper. Algorithm 4 represents a new way to determine the set of core reactions, which used to be a manual step in the old workflow. Algorithm 4 can also be used to refine the core set after reactions are added as a consequence of the External Labeling Variability Analysis (ELVA)).

**Figure 5:**
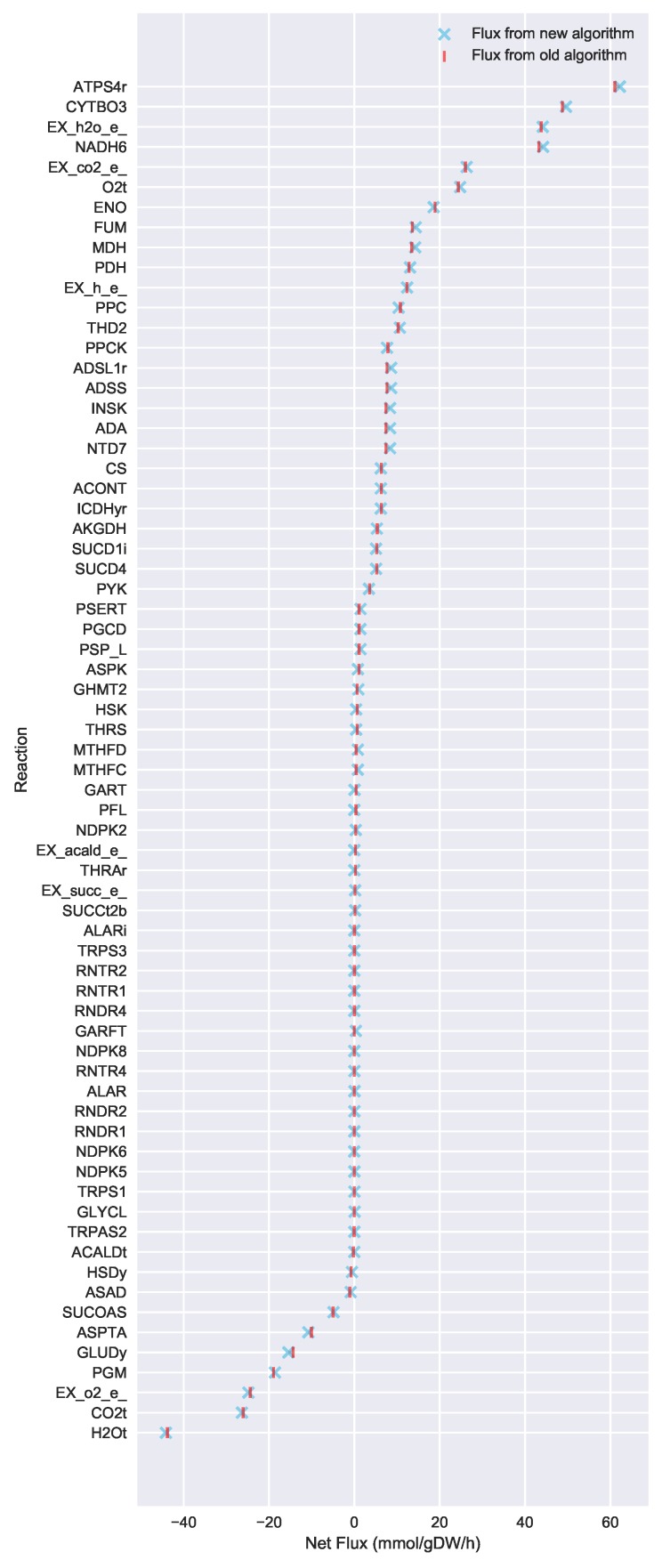
Comparison of 2S-${}^{13}$C MFA fluxes computed using the new vs. old Limit Flux to Core algorithms. Algorithm 2 fluxes are shown in red, whereas the new Algorithm 3 fluxes are shown in sky-blue. Only non-futile cycle fluxes from the iJR904 *E. coli* genome scale model, which were changed by at least 0.01 mmol·gDW${}^{-1}$·h${}^{-1}$ between the two results are shown [36]. Fluxes were computed with the jQMM software tool using the previously published Toya 2010 et al. wild-type 5 h data [31,37]. The final fluxes calculated through 2S-${}^{13}$C MFA are nearly the same using either algorithm. The new algorithm, however, is more systematic and faster since it only requires a single linear optimization problem. Confidence intervals obtained through ${}^{13}$C Flux Variability Analysis [23] can be found in Supplementary Figures S1 and S2.

**Figure 6:**
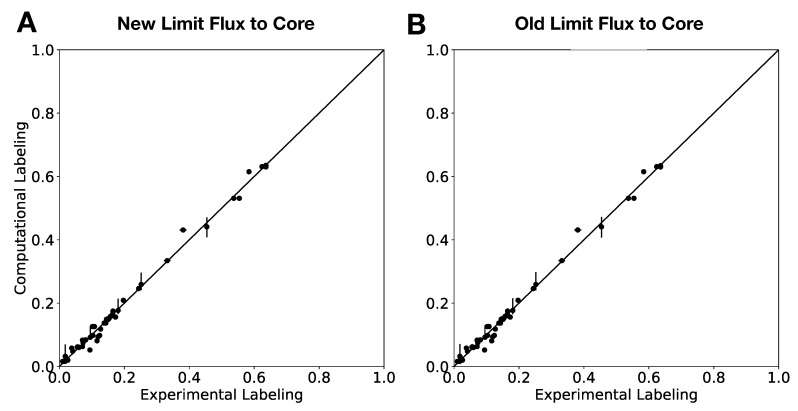
External Labeling Variability Analysis (ELVA) for new (Algorithm 3) and old (Algorithm 2) “Limit flux to core” algorithms shows similar results. The ELVA shows the maximum possible computational error incurred by not adding all reactions to the core, and is used to determine if the core set is complete enough for 2S-${}^{13}$C MFA to be self-consistent. The similarity of both plots shows that both algorithms produce similar results for this important part of the method.

To further test the effect of the new algorithm on the overall flux calculation, we computed 2S-${}^{13}$C MFA flux profiles for the full genome-scale model using the old “Limit flux to core” Algorithm 2 and the new one (Algorithm 3, see Figure 4 and Figure 2 in the original publication [23]). Even though the core stays the same, flux results may change due to the different flux bounds (Figure 3). We used the jQMM library [31] for this purpose, where the “Limit flux to core” step given by Algorithm 2 was substituted by Algorithm 3. After futile cycles have been removed from flux results, only minor differences remain (Figure 5). Furthermore, the External Labeling Variability Analysis (ELVA), showing the impact of reactions not included in the core set on the measured labeling, are very similar (Figure 6). Overall, this demonstrates that, while Algorithm 3 improves the overall modeling workflow by making it more consistent with the bow tie approximation, previously published 2S-${}^{13}$C MFA modeling results may still be regarded as reasonably accurate.

### 3.2. Improving Core Metabolism Boundaries Using Simulated Annealing

**Figure 7:**
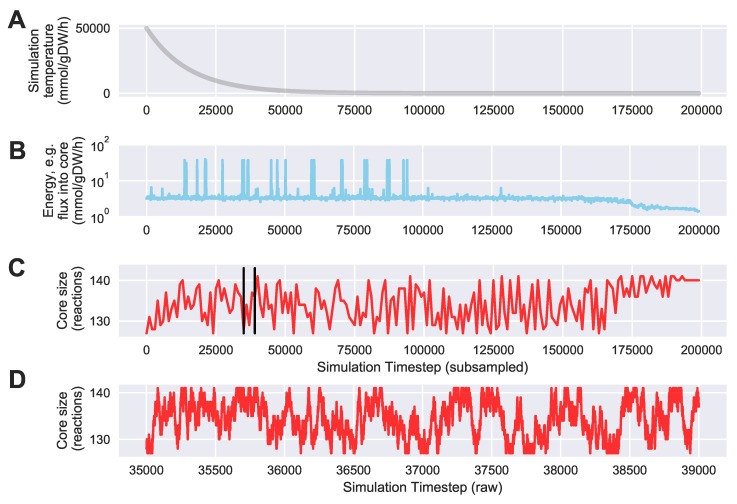
Temperature, energy, and core size during and example Simulated Annealing run to automatically identify an expanded core that minimizes the required sum of fluxes into core metabolism from peripheral metabolism. We started with an example core containing 127 reactions from central carbon metabolism in the iJR904 *E. coli* genome scale model, using exchange and biomass fluxes from the previously published Toya et al. (2010) wild-type 5 h data [36,37]. We ran the simulation for 200,000 steps using an exponentially decaying annealing schedule starting at a “temperature” of 50,000 mmol·gDW${}^{-1}$·h${}^{-1}$ and ending at 0.01 mmol·gDW${}^{-1}$·h${}^{-1}$. The sum of fluxes into core metabolism was reduced from a starting “energy” of 3.19 mmol·gDW${}^{-1}$·h${}^{-1}$ to a final value of 1.20 mmol·gDW${}^{-1}$·h${}^{-1}$. Shown here is the best (lowest flux into core) result over 64 simulations each started with a different random seed; (**A**) the annealing schedule “temperatures” over the course of the simulation; (**B**) a semilogarithmic plot of the “energy level” of the simulation at each iteration, defined as the sum of fluxes into core metabolism as computed with Algorithm 3, subsampled every 100 steps; (**C**) the size of the core in reactions, subsampled every 1000 steps. Vertical black lines indicate the zoomed in timestep range that is used for panel (**D**); (**D**) the size of the core in reactions, zoomed in from part (**C**), from simulation timestep 35,000 to timestep 39,000 without any subsampling.

**Figure 8:**
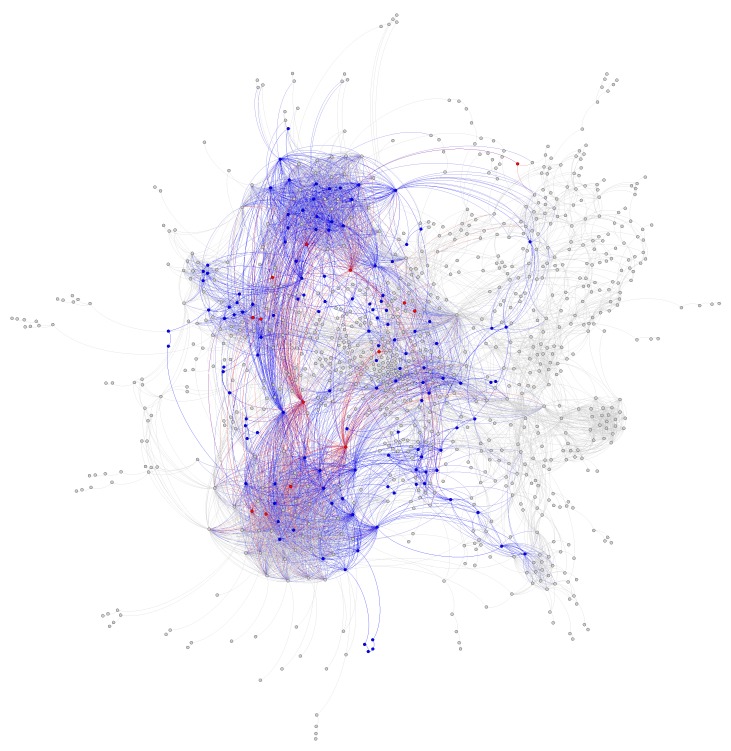
Initial and expanded core metabolism network from Simulated Annealing example run. Shown is the iJR904 *E. coli* genome scale model where each node is a reaction, and each pair of nodes are connected by an edge if they share a common “non-currency” metabolite in their reactants or products. Nodes that were not part of the largest connected component (e.g., reactions not connected to the rest of the graph by non-currency metabolites) are not shown. The initial reaction core is shown in blue, with the new reactions added by the Simulated Annealing run shown in red. All other reactions in the genome scale model (e.g., peripheral metabolism) are shown in gray. Note that several of the added reactions bridge two distinct highly connected clusters in core metabolism, and therefore should be included as part of the core per the bow tie approximation. Graph layout was performed via the Gephi software tool using the ForceAtlas2 force-directed layout algorithm [38,39]. This algorithm was informed only by connectivity, and not influenced by node category (color).

To evaluate the ability of Algorithm 4 to automatically identify an “improved” core with a lower sum of fluxes into core metabolism from peripheral metabolism, we ran a simulation starting with the same 127 core reactions from the iJR904 *E. coli* genome scale model mentioned above. We also used the same exchange and biomass fluxes from Toya et al. (2010) mentioned above. The initial “energy” (i.e., sum of minimized fluxes into core metabolism) for this starting core set was 3.19 mmol·gDW${}^{-1}$·h${}^{-1}$. After running 64 parallel simulations of 200,000 steps, we found a “lowest energy” core set of 141 reactions with a substantially reduced “energy” of only 1.20 mmol·gDW${}^{-1}$·h${}^{-1}$. We used an exponentially decaying annealing schedule starting at a “temperature” of 50,000 mmol·gDW${}^{-1}$·h${}^{-1}$ and ending at 0.01 mmol·gDW${}^{-1}$·h${}^{-1}$. Figure 7 shows a trace of the temperature, energy, and core size (in reactions) over the entire simulation. To reduce the search space, we bounded the solution between a minimum of the 127 reactions in the initial core, and a maximum of 13% of the entire genome scale model, which was equal to the final core size of 141 reactions. As shown in Figure 7C,D, the trace shows excellent mixing over the solution space at high temperatures, whereas Figure 7B shows that the simulation effectively stopped exploring very high energy solutions approximately halfway through the simulation. Figure 8 shows a visual comparison of the initial and final core set, overlaid on a graph representing the entire *E. coli* metabolic network, as described in the figure caption. This graph shows that the initial *E. coli* core contains many reactions divided into two somewhat distinct clusters (upper left and lower left). The simulation largely identified new reactions centered in the “bridge” between these two clusters, which carry flux out of, and back into, the initial core, and therefore should be included inside the core metabolism reaction set. Note that this result is dependent on defining a set of “currency metabolites” that are widely exchanged in metabolic reactions throughout the cell, but do not contribute any carbon (directly or indirectly) to experimentally measured metabolite mass isotopomer distributions. We define a default set of currency metabolites in our Python implementation, but this set should be recomputed for each individual ${}^{13}C$ modeling experiment, based on the metabolites being measured. Overall, this result demonstrates the ability of Algorithm 4 to automatically expand a set of core metabolism reactions, to produce an improved core which better satisfies the bow tie approximation.

## 4. Discussion

In this paper, we have provided systematic methods to delineate how the bow tie approximation takes form within genome-scale models. We have constructed an improved method to constrain metabolic fluxes so as to limit flux into core metabolism, and have created a method to identify the boundaries of core metabolism. The practice of assuming that certain reactions do not affect metabolite labeling is used even in the case of genome-scale ${}^{13}$C MFA (e.g., beta-oxidation, nucleotide salvage pathways), although for a much larger core set [16]. The method presented here can be applied to systematize and make this practice more rigorous.

The new Limit Flux to Core algorithm described in this manuscript computes substantially lower flux limits for reactions flowing into the core while maintaining consistent predicted genome scale fluxes constrained by ${}^{13}$C data. This new algorithm is faster because it does not depend on sequential execution, as flux bounds are simultaneously minimized via linear programming. Additionally, this algorithm is more efficient and does not rely on ad hoc processes such as trial and error and arbitrary cutoff values as the original Limit Flux to Core algorithm does. This provides an improved understanding of the theoretical process of Two-Scale ${}^{13}$C Metabolic Flux Analysis, as it more closely follows the underlying biological assumption that little flux flows from the periphery to the core.

Furthermore, although we have reworked how flux bounds are determined, final flux predictions from the full 2S-${}^{13}$C MFA procedure are consistent with predicted fluxes before changes to the Limit Flux to Core algorithm, where the set of core metabolic reactions is fixed. Figure 5 shows predicted fluxes for only the reactions that have different predicted fluxes between different Limit Flux to Core algorithms, with only marginal predicted flux differences for a handful of reactions.

The Simulated Annealing algorithm described here identifies core boundaries that have low net flux into the core. A possible downside to this approach is that it could inappropriately set low bounds for a boundary reaction that has a physiologically high bidirectional flux but low net flux. In most such cases, the modeling of mass isotopomer distributions in core metabolism would fail to fit accurately to experimental mass isotopomer data, and the user would have to manually add reactions with inappropriate bounds to the core set. This process could be automated in the future with a model for bidirectional fluxes in peripheral metabolism, but remains unaddressed in this manuscript given the technical challenges in rapidly performing non-convex global optimization on large dimensional problems and the lack of reliable characterizations for the carbon atom transitions for reactions in peripheral metabolism.

Lastly, decades of metabolic flux analysis on *E. coli* or other model organisms has resulted in a determination of core metabolic networks that give reasonable flux results and that satisfy the bow tie approximation well. However, for new organisms, model organisms under uncommon laboratory conditions (e.g., a different carbon source), or novel genetically engineered strains, no reasonable core reaction set is known. For these cases, the Simulated Annealing algorithm described here provides a general method for automatically identifying an improved set of core reactions that better satisfies the bow tie approximation. This method and the open source implementation we provide is of general use for the ${}^{13}$C MFA, 2S-${}^{13}$C MFA and FBA communities, as it has the potential to substantially reduce the manual effort required for ${}^{13}$C flux modeling in new organisms, and/or new experimental conditions.

## Supplementary Materials

The following are available online at www.mdpi.com/2218-1989/8/1/3/s1. Figure S1. Comparison of 2S-${}^{13}$C MFA flux bounds computed using the new vs. old Limit Flux to Core algorithms. Figure S2. Comparison of 2S-${}^{13}$C MFA flux bounds computed using the new vs. old Limit Flux to Core algorithms with the most likely flux shown.

## Abbreviations

The following abbreviations are used in this manuscript:

FBA	Flux Balance Analysis
${}^{13}$C MFA	${}^{13}$C Metabolic Flux Analysis
2S-${}^{13}$C MFA	Two-Scale ${}^{13}$C Metabolic Flux Analysis
