# Supplementary figures and images for: Constraining Genome-Scale Models to Represent the Bow Tie Structure of Metabolism for 13C Metabolic Flux Analysis
Source: Metabolites. 2018 Jan 4;8(1):3. doi: 10.3390/metabo8010003 (PMC5875993; doi:10.3390/metabo8010003)

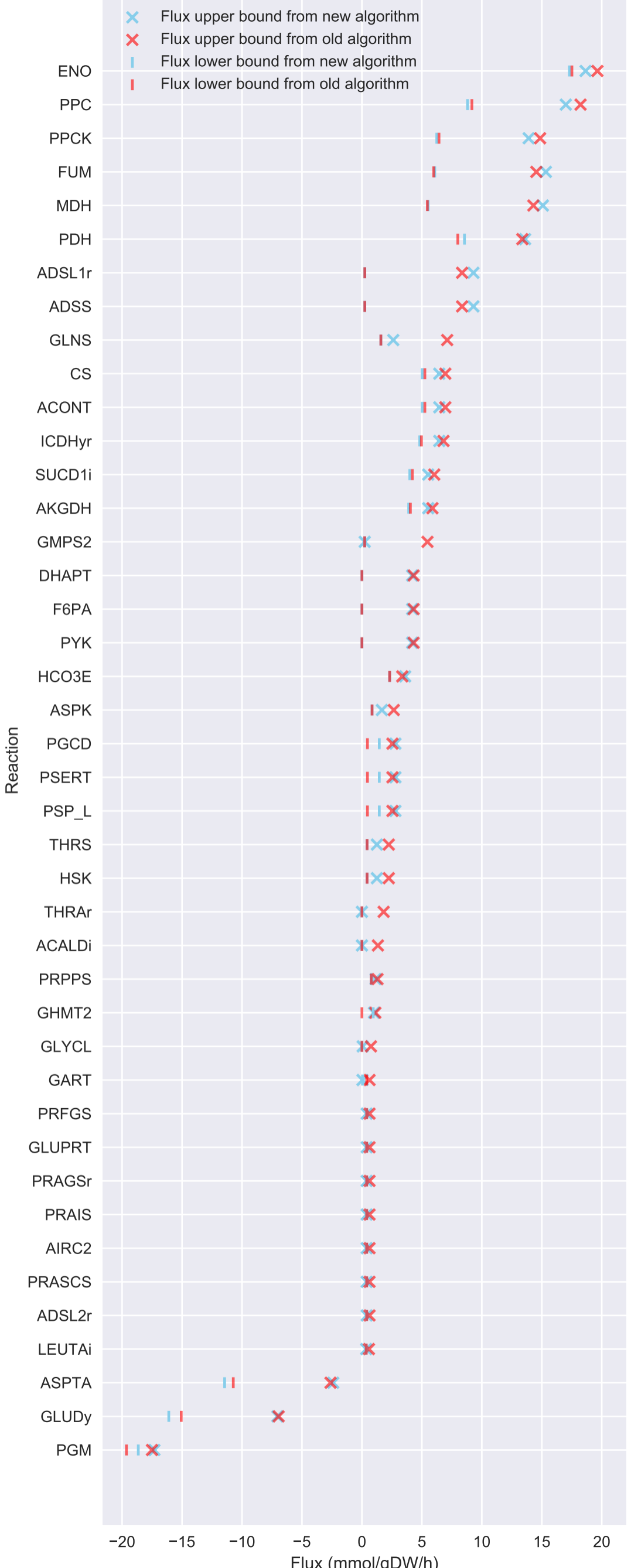

Supplement: Supplementary file 1 [file metabolites-08-00003-s001.zip › metabolites-246538-supplementary/Figure S1.pdf]

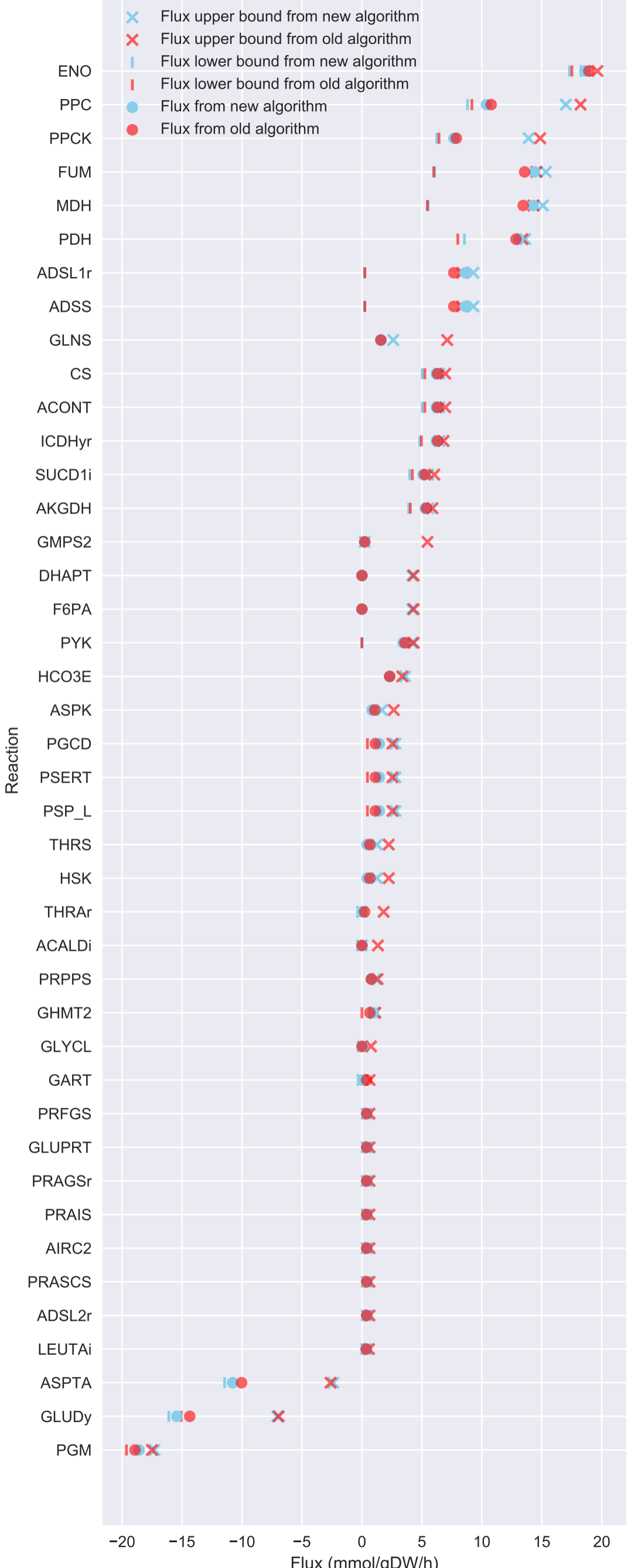

Supplement: Supplementary file 1 [file metabolites-08-00003-s001.zip › metabolites-246538-supplementary/Figure S2.pdf]
